# Seasonal Variations in Heavy Metal Concentrations in Mussels (*Mytilus chilensis*) from Southern Chile: Health Risk Implications Associated with Their Consumption

**DOI:** 10.3390/foods14060916

**Published:** 2025-03-07

**Authors:** Ociel Muñoz-Fariña, Analese Roman-Benn, Carmen Lopez-Joven, Luisbel González-Pérez de Medina, María Cristina Ravanal

**Affiliations:** 1 Food Science and Technology Institute (ICYTAL), Faculty of Agricultural and Food Sciences, Universidad Austral de Chile, Campus Isla Teja, Valdivia 5090000, Chile; maria.ravanal@uach.cl; 2 Food Policy Division, Ministry of Health, Georgetown 05924, Guyana; analeseroman@gmail.com; 3 Institute of Veterinary Preventive Medicine, Faculty of Veterinary Sciences, Universidad Austral de Chile, Campus Isla Teja, Valdivia 5090000, Chile; carmen.lopez@uach.cl; 4 Laboratory of Biomaterials, Department of Chemical Engineering, Faculty of Engineering, University of Concepción (UdeC), Concepción 4030000, Chile; luisbgonzalez@udec.cl

**Keywords:** *Mytilus chilensis*, bioaccessibility, Chile, arsenic, lead, cadmium

## Abstract

*Mytilus chilensis* is considered an important food source for the Chilean population and represents a considerable fraction of its aquacultural production, mainly in southern Chile’s coastal regions. This study aimed to assess the concentrations of total arsenic (tAs), lead (Pb), and cadmium (Cd), their bioaccessibility, and associated health risks in *M. chilensis* from the Valdivia River Estuary (VRE) in the Los Ríos Region and the Reloncaví Fjord (RF) in the Los Lagos Region. The metal concentrations were quantified using Inductively Coupled Plasma–Optical Emission Spectrometry (ICP-OES). The concentrations of tAs, Cd, and Pb were 6682 ± 2018, 1592 ± 742, and 1208 ± 639 ng/g d.w., respectively. Variations in the metal concentrations were observed across areas, months, and sampling points but remained below national and international limits. No correlation was found between the metal concentrations and environmental parameters. The bioaccessibility percentages were tAs (68 ± 10%), Cd (45 ± 21%), and Pb (15 ± 4%). The tAs, Pb and Cd levels in *M. chilensis* from southern Chile do not represent a risk to human health.

## 1. Introduction

*Mytilus chilensis* is a native species also known as “chorito”, “quilmahue” or “Chilean blue mussel”; it is a bivalve of the *Mytilidae* family found on the Chilean coast from Arica (far north of the country) to Tierra del Fuego (far south of the country) in intertidal and subtidal environments at depths of up to 25 m [1,2]. This species is the main bivalve cultivated in Chile, representing a considerable fraction of the aquacultural production in the country, having achieved a production of 375,700 tons in 2021, being only surpassed by the Atlantic salmon [3,4]. These products have positioned Chile as the second-largest mussel producer in the world and the main exporter of *M. chilensis* [5].

Mollusk aquaculture in Chile is favored due to its numerous estuaries and bays, especially in the southern coastal areas of the country [6]. Ninety-nine percent of the total commercial production of *M. chilensis* takes place in the south of Chile, specifically in the Los Lagos Region [3,7]. It is also an important food source for the population due to its high biological protein content, which provides many essential amino acids, and its high content of essential fatty acids such as long-chain omega-3 fatty acids, which promote cardiovascular health [6,8]. Additionally, bivalves of the genus *Mytilus* are used as indicators of the aquatic environmental quality due to their capacity to bioaccumulate metals without any signs of alteration since they are sessile filter feeders that can tolerate a wide range of environmental conditions [9,10,11]. In this context, metals accumulated by bivalves and other aquatic animals can be transferred to human consumers through biomagnification and pose a risk to public health [12,13,14]. Contaminants such as arsenic, lead, and cadmium in foods, even in small amounts, can cause serious health problems in humans due to their toxic and accumulative nature [15,16,17].

Arsenic (As) is a metalloid of great environmental and public health importance, characterized by its ability to exist in four oxidation states: −III, 0, +III, and +V; this allows it to form a variety of organic and inorganic compounds [18]. Inorganic arsenic compounds are considered highly toxic and are classified as probable carcinogens by the International Agency for Research on Cancer (IARC) [19]. Chronic exposure to this toxic metal has been associated with a variety of diseases, including several types of cancer, cardiovascular disease, and adverse effects on child development [20,21,22,23]. A review of the inorganic arsenic (iAs) levels in mussels [24,25,26,27,28,29,30,31], based on more than a thousand samples analyzed in various studies, found that only four samples exceeded 10% of tAs, representing less than 0.4% of the total examined. Based on these findings, 10% of total arsenic (tAs) being equivalent to iAs is a reasonable estimate.

Lead exists in both inorganic and organic forms [32,33,34]. The presence of lead in the environment is of concern because it can be found in foods such as vegetables, seafood, and fish, and can contaminate drinking water through pipes and industrial waste. The inorganic form of lead is not metabolized in the liver, while ingested organic lead (from gasoline additives) is almost completely absorbed and metabolized in this organ [35,36,37]. Exposure to this contaminant can have serious health effects, affecting the central nervous system, kidney function, and hemoglobin production, potentially leading to problems such as encephalopathy, hypertension, and reproductive disorders [38,39].

Cadmium is a heavy metal that occurs naturally in very low concentrations, approximately in the order of 10^−6^ ppb, in the Earth’s crust, and is rarely found in minerals, generally being formed as a by-product of the extraction of other metals such as zinc, copper, and lead. However, its environmental and public health significance lies in its potential for contamination [40,41]. Exposure to cadmium occurs primarily through the ingestion of contaminated food and water. The regulation of this metal is critical, with institutions such as the FAO/WHO and EFSA setting limits and recommendations to protect the public from cadmium exposure in food [42].

Given that not all metals present in food are absorbed by the human body during the process of digestion, it would be inaccurate to determine the risks associated with human health only by their concentrations found in food and not by their bioaccessible fractions [43,44,45]. To exert a toxic effect on a specific tissue or organ, heavy metals must first be extracted from the food matrix. Therefore, bioaccessibility refers to the fraction of a nutrient, toxin, or other substance that, when released from its matrix, becomes soluble in the gastrointestinal environment by the digestive juices, and thus becomes available for absorption into the systemic circulation through the intestinal wall [46,47,48]. Although in vivo digestion in humans and animals would be ideal, it is technically difficult, costly, and limited by ethical issues when dealing with potentially harmful substances, so in vitro models are designed to closely mimic the physiological processes that occur during human digestion [49,50]. Among the wide range of gastrointestinal models that exist, static models are fast, simple, and cost-effective. They are widely used and have numerous advantages, mimicking the biochemical processes that occur in the gastrointestinal tract, particularly in the oral, gastric, and intestinal phases [49,51]. Its main applications include the hydrolysis of macronutrients, and the solubility, release, and bioaccessibility of different compounds from simple food matrices [46,52].

Mussels are consumed whole, with their internal organs, as opposed to fish, of which only the muscles are consumed; therefore, once eaten, they increase the risk of contamination by toxic substances in humans [14,52]. Therefore, this study aims to quantify the concentration of arsenic, lead, and cadmium in specimens of *Mytilus chilensis* from southern Chile to evaluate their bioaccessibility and the health risks associated with their consumption.

## 2. Materials and Methods

### 2.1. Reagents

Deionized water (Merck, Darmstadt, Germany catalog number: 848333) was used to prepare reagents and standards. All chemicals were of pro-analysis quality or better. Commercial standard solutions of As, Pb, and Cd (1000 µg/mL) (Merck, Darmstadt, Germany) were used.

### 2.2. Study Area

**Figure 1:**
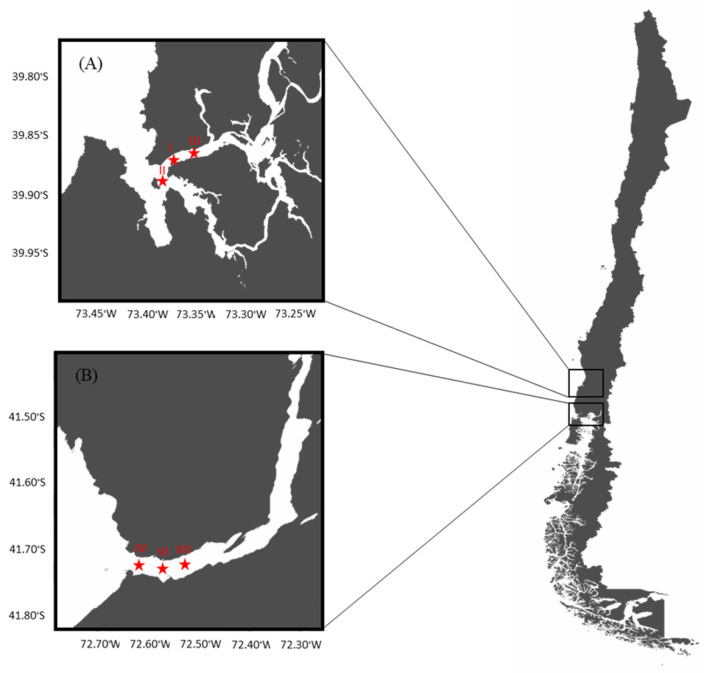
Map of sampling sites and points: (**A**) Valdivia River Estuary and (**B**) Reloncaví Fjord.

A longitudinal study was conducted in two selected areas performing the natural extraction of the bivalves, *Mytilus chilensis*, on the southeastern Pacific coast of Chile: the Valdivia River Estuary (VRE) in the Los Ríos Region and the Reloncaví Fjord (RF) in the Los Lagos Region. Three sampling points were established in each area of extraction (Figure 1).

The VRE is considered a local natural mussel extraction zone that plays an important role in tourism. This zone has a tidal range of less than 2 m and the mussels are found in the subtidal zone, being less exposed to direct solar radiation, which implies lower internal temperature variations. The coordinates of the sampling points were point I (39°52′33′′ S, 73°23′08′′ W), point II (39°53′39′′ S, 73°23′10′′ W), and point III (39°52′10′′ S, 73°21′19′′ W).

On the other hand, the RF is located approximately 115 km from the city of Puerto Montt. Together with Chiloe Island, they are considered the main mollusk production areas at the national level. This area is influenced by the ocean and by the rivers Petrohué, Cochamó and Puelo, and the glaciers. The tidal range is between 6 and 7 m and the mussels are found in the intertidal zone, exposed to solar radiation for several hours. The coordinates of the sampling points were point IV (41°42′33′′ S, 72°36′60′′ W), point VI (41°42′37′′ S, 72°36′32′′ W), and point VII (41°42′37′′ S, 72°36′06′′ W). Environmental parameters such as the temperature (°C), pH, conductivity (mS/cm), and salinity (ppt) of water (YSI Model 30 Salinity, Conductivity and Temperature System, Xylem Inc., Yellow Springs, OH, USA) for each sampling point, at two depths of the water column (0–2 m and 2–8 m), were recorded monthly.

### 2.3. Sample Collection

*Mytilus chilensis* samples were collected between January and December 2017 from the two extraction areas. In total, 58 samples were collected: 29 from the VRE and 29 from the RF. The soft parts were extracted from the shells with plastic tools to avoid possible metal contamination and were frozen in resealable polyethylene Ziploc^®^ bags until further analysis. Homogenization was performed with an immersion blender (Oster^®^ 2612, Atlanta, GA, USA).

### 2.4. Determination of Total Arsenic (tAs), Lead (Pb) and Cadmium (Cd)

The analyses were performed in triplicate by dry mineralization according to the methodology of the Association of Official Analytical Chemists (AOAC) [53]. Of the previously homogenized samples, 2.00 ± 0.001 g was wet-weighed into beakers of 100 mL, and the following reagents were added: 5 mL of distilled water, 1 mL of MgO (7.4% *w*/*v*), 5 mL de H_2_O_2_ (30% *v*/*v*) and 20 mL of concentrated HNO_3_. The treated samples were placed on a heating plate (Oster^®^, Atlanta, GA, USA) at 80 °C, where they were subjected to digestion until a volume of approximately 10 mL of partially digested sample was achieved. Subsequently, 20 mL of concentrated HNO_3_ was added and the digestion continued until complete dryness was achieved.

The samples were then placed in a muffle furnace (model: Type 6000 FURNACE Thermolyne, Waltham, MA, USA) whose temperature gradually increased until reaching 450 °C and remained under those conditions for 12 h to obtain white ash. If organic matter was still present after the mineralization process, 10 mL of HNO_3_ (50% *v*/*v*) was added to the sample and it was returned to the heating plate until dryness was achieved and the mineralization was repeated in the muffle furnace. After mineralization, 1 mL of ultra-pure water and 1 mL of concentrated HCl were added to dissolve the ash; it was then filtered with Whatman™ No. 1 filter paper and HCl (10% *v*/*v*) was used to reach a final volume of 25 mL. The samples were placed in falcon^®^ plastic flasks (50 mL) and refrigerated at 4 °C until further analysis. The concentrations of tAs, Pb, and Cd were expressed in dry weight.

### 2.5. In Vitro Gastrointestinal Digestion

After the determination of the tAs, Pb, and Cd concentrations, the three samples with the highest concentrations of these metals were mixed and subjected to an in vitro gastrointestinal digestion assay to analyze the bioaccessibility of those metals. The samples were analyzed in their raw form to evaluate the release of the metals from their matrix since they are frequently consumed raw or undercooked in Chilean culture.

The in vitro gastrointestinal digestion assay simulated the physiological conditions in the five stages of the digestive process: the oral phase (mouth), gastric phase (stomach), and gastrointestinal digestion phases (duodenum, jejunum, and ileum); this was performed according to a procedure described by Ah-Hen et al. [54]. To simulate the oral phase, 50 g of the sample was mixed with 80 mL of deionized water and 12 mL of artificial saliva (521 mg of NaHCO_3_, 88 mg of NaCl, 48 mg of KCl, 44 mg of CaCl_2_·2H_2_O,104 mg of K_2_HPO_4_, 216 mg of mucin and 10 mg/mL of α-amylase at a pH of 6.8). This mixture was placed in a Stomacher^®^ bag and placed in a paddle homogenizer (iUL instruments, Barcelona, Spain) for 1.5 min. Once the mixture was homogenized, a sample of 10 mL was taken. To simulate the gastric phase, the mixture was placed in a 250 mL Schott^®^ flask, and 10 mL of pepsin (45 mg/mL) from porcine gastric mucosa (Sigma Aldrich, ≥250 units/mg solid, St. Louis, MO, USA), dissolved in ultrapure water, was added to it; the pH was adjusted to 2.0 with HCl 6 N and the sample was incubated in a thermoregulated bath (model: ZHWY-110X, Zhicheng Co., Shanghai, China) at 37 °C and kept in agitation for 2 h at 200 rpm to simulate peristaltic mixing.

After that time passed, a sample of 10 mL was taken. To simulate the small intestine (duodenum), the mixture was then adjusted to a pH of 6.5 with NaHCO_3_ 0.5 N; then, 10 mL of a mixture of pancreatin (180 mg/mL) and bile salts (2.25 mg/mL) was added. The mixture was homogenized, and a sample of 10 mL was taken. The flask with the mixture was then incubated again at 37 °C for a 2 h agitation at 200 rpm. After the first hour of agitation, another sample of 10 mL was taken to analyze the jejunum and, after the second hour, another sample of 10 mL was taken to analyze the final section of the intestine, the ileum. The collected samples were centrifuged at 9000 rpm for 10 min in a centrifuge (Hettich Zentrifugen, model: Universal 320R, Tuttlingen, Baden-Württemberg, Germany). Then, the supernatant was ultra-centrifuged at 12,000 rpm for 15 min in an ultra-centrifuge (Boeco, model: M-24^®^, Boeco, Hettich, Germany). The supernatant was then used for the mineralization process previously described to determine the concentrations of tAs, Pb, and Cd it contained.

The bioaccessibility of tAs, Pb, and Cd was calculated using the total concentration of *M. chilensis* found in the samples according to Equation (1) [55]:

(1) $$Bioaccessibility\;of\;Metal\;\%=\frac{Bioaccessible\;metal}{Total\;metal}\times 100$$

The concentrations are indicated on a dry weight (d.w.) basis, except in cases where they were used for comparison with the maximum limits established by the Food Sanitary Regulation (RSA), Contaminants in the Food Chain Panel, and point FAO/WHO Expert Committee on Food Additives (JEFCA) for each metal and when they were used to calculate the Estimated Daily Intake (EDI) and percentages of bioaccessibility after the in vitro gastrointestinal digestion assay.

### 2.6. Calibration Curve and Sample Reading

A standard for each of the metals (t-As, Pb, and Cd) was prepared from their corresponding commercial standard solution of 1000 µg/mL (Merck, Darmstadt, Germany), respectively. The concentrations of tAs, Pb, and Cd in the samples were determined by Inductively Coupled Plasma–Optical Emission Spectrometry (ICP-OES) (PlasmaQuant 9100 Analytic Jena—Analytik Jena GmBH, Jena, Germany). The instrumental conditions and analytical parameters are shown in Table 1. The reading was performed by bringing the equipment to an optimum working condition and calibrating it using the calibration curve defined for each metal specifically, with a minimum acceptable adjustment of 99.95%.

**Table 1:** Operating conditions and analytical parameters for inductively coupled plasma–optical emission spectrometry (ICP-OES).

Parameters	tAs	Pb	Cd
Wavelength spectrum (nm)	193.698	220.353	226.502
Detection limit (μg/L)	0.121	0.065	0.004
Detection	axial		
Line gas pressure (psi)	6		
Gas purity	Argon (99.99%)		
Gas flow (L/min)	0.50		
Wavelength spectrum (nm)	6		
Nebulizer			
Pressure (bar)	3.20		
Carrier gas flow (L/min)	0.50		
Pumping speed (mL/min)	4.00		
Integration period (s)	65.00		
Wash period between samples (s)	20.00		
Analytical parameters			
Accuracy (Recovery Percentage)	114.7	103.6	116.8
Precision (Coefficient of variation)	1.76	1.84	0.46
Instrument detection limit (IDL) (ng/mL)	0.121	0.065	0.004
The method detection limit (MDL) (ng/mL)	0.302	0.161	0.009

### 2.7. Human Health Risk Assessment

For the evaluation of the carcinogenic risk posed by the ingestion of foods contaminated with heavy metals, the strategy described by Saha et al. was applied [56].

#### 2.7.1. Estimated Daily Intake (EDI)

(2) $$EDI=\frac{\left(F\cdot Cm\right)}{W}$$

where *F* is the consumption of *Mytilus chilensis* (18.6 g/day) [22], Cm is the metal concentration in the food sample (μg/g w.w.), and W is the body weight of a 70 kg adult [22].

##### Non-Carcinogenic Risk: Target Hazard Quotient (THQ)

The Target Hazard Quotient (THQ) is used to express the risk of non-carcinogenic effects and enables an assessment of the potential human health risks associated with long-term exposure to heavy metals in food, in combination with the EDI and the oral reference dose (RfD in mg/(kg bw day)), as shown in Equation (3).

(3) $$THQ=\frac{EDI}{RfD}$$

The oral RfD represents an estimate of the daily exposure to which the human population can be continually exposed over a lifetime without an appreciable risk of harmful effects. The RfDs are based on values of 0.0003, 0.0035, and 0.001 mg/(kg bw day) for iAs, Pb, and Cd, respectively [55].

If the THQ value is less than 1, the exposed population should not experience any adverse health hazards. On the contrary, the exposed population may experience non-carcinogenic health risks if the THQ is equal to 1, and as the value increases, so does the probability. The method for determining THQ was provided in the USEPA Region III risk-based concentration table [22]. The THQ calculations were performed because the ingestion dose is equal to the absorbed contaminant dose and the average body weight of an adult is 70 kg [55].

Studies have shown that exposure to two or more contaminants can have an additive and/or interactive effect. Thus, in the present study, the cumulative health risks were evaluated by summing the THQ values of individual metals and expressing them as the Total Target Hazard Quotient (TTHQ) (also called hazard index, HI), calculated as shown in Equation (4).

(4) $$TTHQ=\sum_{i=1}^{n}{THQ}_{i}$$

A TTHQ value greater than 1 generally indicates the potential for adverse human health effects and suggests the need to undertake a higher level of research or possibly remedial action [22].

##### Carcinogenic Risk (CR)

For the determination of the carcinogenic risk (CR), the methodology described by Saha and Zaman was applied [57]. The CR indicates the incremental probability of an individual developing cancer throughout their lifetime due to exposure to a potential carcinogen. Equation (5) was used to calculate the CR.

(5) CR=CSF·EDI

The CSF is the carcinogenic slope factor and its stipulated values are 1.5, 0.5, and 6.1 milligrams per kilogram of body weight per day (expressed in units of reciprocal dose (mg/(kg day))^−1^) for iAs, Pb, and Cd, respectively [58]. The acceptable lifetime CR is 10^−4^ to 10^−6^ (average lifetime risk of developing cancer is 1 in every 100,000 persons) [55].

Additionally, all parameters related to health risk assessment (EDI, THQ, and CR) were estimated assuming different iAs/tAs ratio values to provide more information on the safety of mussel consumption in southern Chile. The iAs/tAs values used in this study were 5, 10, 20, 30 and 50%.

### 2.8. Validation of Methodology

The validation of the methodology allows for the verification of whether the method used was adequate for the analytical problem being solved and if the data is indeed truthful. For this purpose, different parameters such as accuracy, precision, instrumental detection limit, and methodology detection limit were determined.

The accuracy (Recovery%) and precision (Coefficient of variation%) of the methodology were determined in the following manner: six replicates of a sample were selected, and a determined amount of the analyte was added to three of them. The corresponding analytical procedure was carried out and the metal concentrations were quantified. Finally, the results obtained were compared between the replicates with the addition of the standard and those without it.

The instrument detection limit (IDL) was determined by analyzing six blanks, calculating the standard deviation of the basal concentrations that they possessed, and multiplying it by three (the factor that allows for a 95% assurance that the signal is significantly different from the base limit). To determine the method detection limit (MDL), the instrument detection limit (IDL) was multiplied by the dilution factor (DF) and divided by the average weight of the samples (p).

The percentage of recovery was found to be above 100% for all three metals and coefficients of variation of less than 2% were obtained, demonstrating that the mean is representative of the sample and the high sensitivity of the methodology used in the analysis. The blanks used underwent the entire analytical procedure and were performed in triplicate; no traces of As, Pb, and Cd were detected, which ruled out possible contamination by the reagents used. The concentrations in the investigation are indicated on a dry weight (dw) basis, except in cases where they were used for comparison with the maximum limits established by the RSA, CONTAM Panel, and JEFCA for each metal and when they were used to calculate the percentages of bioaccessibility after the in vitro gastrointestinal digestion assay.

### 2.9. Statistical Analysis

For the statistical analysis, a correlation analysis and simple analyses of variance were performed to compare the concentrations of tAs, Pb, and Cd in the samples using the STATGRAPHICS Centurion XV software (version number 15.2, Statgraphics Technologies, Inc., The Plains, VA, USA). Significant differences were corroborated with a multiple-range analysis using Tukey’s test. The adequacy of the intake of each of the metals was calculated using the values of the average daily consumption of *M. chilensis* in southern Chile according to the information collected from the study of Muñoz et al., multiplying it by the concentration of each metal in the samples [22].

## 3. Results and Discussion

### 3.1. General Comparison of the Metals Studied: Total Arsenic, Lead, and Cadmium in Specimens of M. chilensis

The average concentrations of tAs, Pb, and Cd in the mussel samples were 6421 ± 1937, 1202 ± 588, and 595 ± 807 ng/g dw, respectively. These values are presented in Table 2, accompanied by a comparative literature review summarizing the various studies conducted worldwide.

The average concentration of tAs in the samples analyzed in this study was higher than the concentrations found in mussels from Slovenia, Croatia, Spain, and Chile in the Serbian market [59], those marketed in markets in the Canary Islands (Spain) that were originally from Galicia, Chile and New Zealand [60], and in specimens of *M. galloprovincialis* from Italy [13]. On the other hand, they were lower than those found in *M. galloprovincialis* from Montenegro [61] and from Turkey [62], and in specimens of *M. chilensis* from Chile [63]. These results can be attributed to the ubiquitous nature of tAs, which makes it readily available in the aquatic environment due to atmospheric deposition, river runoff, and the upwelling of marine sediments, leading to the enrichment of arsenic in marine organisms [31,64].

**Table 2:** Concentrations of tAs, Pb, and Cd (ng/g dry mass) in mussel specimens from different countries worldwide.

Location	Species	tAs (ng/g d.w.)	Pb (ng/g d.w.)	Cd (ng 7 g d.w.)	Reference
Chile	*M. chilensis*	6421 ± 1937	1202 ± 588	1595 ± 807	Present study
Chile	*M. chilensis*	540	330	n.d	[65]
Argentina	*M. chilensis*	n.d	420 ± 360	750 ± 480	[66]
Chile	*M. chilensis*	7480 ± 1720	n.d	1940 ± 300	[22]
Chile	*M. chilensis*	n.d	5260 ± 550	2470 ± 180	[63]
Montenegro	*M. galloprovincialis*	14,700 ± 2100	980 ± 230	840 ± 180	[61]
Algeria	*M. galloprovincialis*	n.d	7490	660	[67]
Italy	*M. galloprovincialis*	942 ± 100	513 ± 290	50 ± 0	[13]
Turkey	*M. galloprovincialis*	9300 ± 3800	7900 ± 7720	1090 ± 880	[62]
Malaysia	*Perna viridis*	n.d	470 ± 140	300 ± 60	[68]
South Africa	*M. galloprovincialis*	n.d	7300	1990	[69]
Serbia	Mixture of mussels	3970 ± 870 ^a^ 1560 ± 360 ^b^	n.d	n.d	[59]
Galicia, Chile and New Zeeland	Mixture of mussels	3697 ± 433 ^a^ 4008 ± 59 ^b^	249 ± 65 ^a^ 177 ± 181 ^b^	281 ± 89 ^a^ 463 ± 306 ^b^	[60]

^a^ Fresh sample and ^b^ Frozen sample. n.d—not determine.

The overall averages of Pb and Cd in the present study are also higher than those found in Galicia [60] and Montenegro [61]. Likewise, they exceed the values found in *M. chilensis* from Chile [65] and from Argentina [66], *Perna viridis* from Malaysia [68], and *M. galloprovincialis* from Italy [13]. However, they are lower than the values found in *M. galloprovincialis* from South Africa [69], in *M. chilensis* from Chile [6], in *M. galloprovincialis* from Turkey [62], and in *M. edulis*, *M.* unguiculatus and *Perna viridis* from China [70]. Other studies showed average Pb levels higher than the results obtained in the present study, but the opposite in the case of Cd [67]. The average Cd levels obtained in the present study are similar to the values recorded in *M. chilensis* from Chile [63].

These results are because the byssus of mussels is more selective and sensitive to variable concentrations of metals (except for Cd) in the environment as opposed to the soft tissue [71]. Lead undergoes an effective transfer process from the soft tissue to the byssus, unlike Cd, which accumulates mainly in the soft tissue. In shellfish, Pb concentrations are higher in the calcium-rich shell than in the soft tissues [13]. Furthermore, the low accumulation rate of Pb compared to that of Cd and As in the marine environment could be because this metal is strongly adsorbed on sediments and suspended particles, which reduces its availability to organisms [72,73,74].

### 3.2. Total Arsenic (tAs) in Specimens of M. chilensis by Study Area

The concentrations of tAs in the samples of *M. chilensis* were determined by the areas, the points, and the months of sampling. About the areas, the samples from the RF (6623 ± 1508 ng/g) were not statistically different from those from the VRE (6218 ± 2278 ng/g) (*p*-value > 0.05).

**Figure 2:**
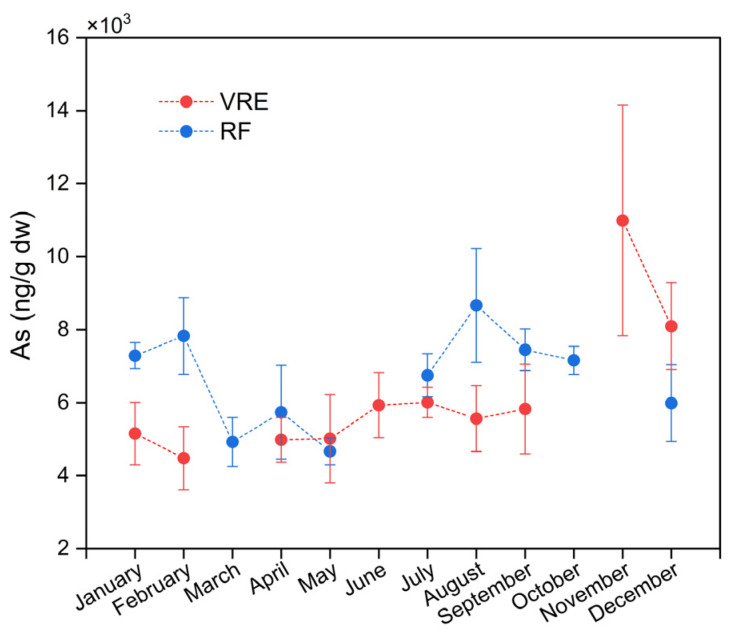
Total arsenic concentrations in *M. chilensis* specimens from the Valdivia River Estuary vs. Reloncaví Fjord according to the month of sampling.

When stratified by study area, it was observed that in the VRE, the samples with the highest concentration of tAs were collected in November (10,992 ± 3158 ng/g) (spring period); meanwhile, the lowest concentration was recorded for the samples collected in February (4480 ± 867 ng/g) (Figure 2). The pattern followed by these results in the VRE coincides with that in a study by Fattorini et al. [75]; in their evaluation of the seasonal, spatial, and interannual (2001–2005) variations in trace metals in *M. galloprovincialis* from the Adriatic Sea, they observed a significant increase of tAs in the samples collected during the winter (11,800 ± 2480 ng/g) to the spring (21,500 ± 15,700 ng/g) of 2002. A study of the same environment performed in Sardinia, Italy, found that samples of *M. galloprovincialis* collected during the spring and summer also contained the highest concentrations of tAs in their study [76].

**Figure 3:**
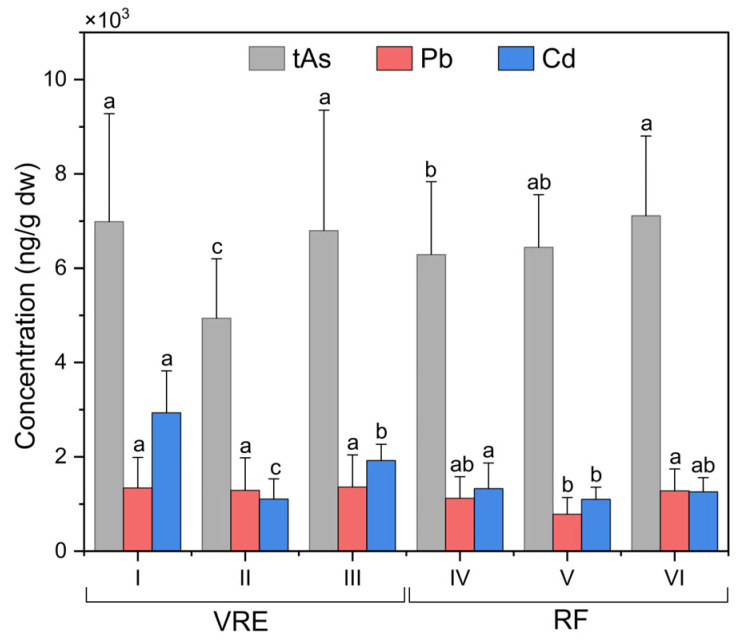
Total arsenic, lead, and cadmium concentrations in specimens of *M. chilensis* from the Valdivia River Estuary (VRE) and the Reloncaví Fjord (RF) according to sampling points. Letters indicate significant differences at a *p*-value < 0.05 for the mean comparison tests for each component.

When comparing by area and points of sampling (Figure 3), the results showed significant differences between the concentration of tAs in the samples collected from points I (6991 ± 2287 ng/g) and III (6799 ± 2555 ng/g), and samples from point II (4941 ± 1257 ng/g) with a lower concentration. In the RF, the samples that contained the highest concentration of tAs were those collected in August (8665 ± 1563 ng/g) followed by those collected in February (7828 ± 1049 ng/g), September (7448 ± 571 ng/g), January (7288 ± 358 ng/g) and October (7160 ± 384 ng/g). Meanwhile, the lowest concentrations were recorded in those collected in March (4923 ± 671 ng/g) and May (4667 ± 369 ng/g) (Figure 2).

A similar seasonal pattern was reported in a study of *M. edulis* transferred to Arcachon Bay, France, where they observed that the samples with the highest concentrations of tAs were collected during the winter, with an annual mean of 15,000 ng/g [77]. Another study of the seasonal variability of the metal and metalloid contents in *M. edulis* from three sites in the Baltic Sea observed that samples from the site Darsser Ort had the highest concentration of tAs (10,210 ng/g) during the winter [78]. This is because the nutritional status of mussels is not optimal during the winter due to a decrease in their food supply during this season; thus, their heavy metal concentrations and weight increase as a result [79].

A study conducted in Rijeka Bay (North Adriatic Sea, Croatia) evaluated the seasonal concentration of tAs in the edible parts of *M. galloprovincialis*. They observed an increase in tAs in mussels harvested during the winter and spring [64], an observation that coincides with the patterns observed in both RF and VRE, respectively. They attributed the higher levels of arsenic observed in the spring to an increase in nutrients that occurs in late winter and early spring due to greater freshwater inputs.

When analyzing the sampling points in the RF (Figure 3), it was observed that the samples collected from point VII (7393 ± 1875 ng/g) were statistically superior to those collected from points VI (6646 ± 1129 ng/g) and IV (6581 ± 1263 ng/g).

The Chilean legislation, through the RSA, still lacks data regarding the maximum allowable limits of arsenic in bivalve mollusks. This coincides with the fact that internationally, the estimated levels of inorganic arsenic (iAs) in mollusks and crustaceans are relatively low when compared to organic species, whose possible toxicity is not known in detail [5].

In the present study, the value of the average daily consumption of seafood (18.6 g/day) in southern Chile according to the study was used to estimate the exposure to As, Pb, and Cd [22]. The EDI of tAs in the samples of *M. chilensis* was 0.32 µg/kg bw/day. However, considering the reduced toxicity of organic arsenic, the measurement of health risks through tAs would not have been adequate, so it was necessary to determine the concentration of iAs in the samples. It was assumed that the iAs presented by the samples did not exceed 10% of the tAs, making the EDI value of iAs 0.032 µg/(kg bw day) [22]. This value is lower than those found at 0.93 µg/(kg bw day) in Brazil [80], 1.32 µg/(kg bw day) in South Africa [81], 2.22 µg/(kg bw day) in China [82], 2.42 µg/(kg bw day) in Spain [83], 8.1 µg/(kg bw day) in Indonesia [84], 9.6 µg/(kg bw day) in Croatia [85] and 119 µg/(kg bw day) in Turkey [62].

The Target Hazard Quotient value associated with the consumption of *M. chilensis* was 0.11; at <1, the Chilean population should not experience any adverse health risks associated with long-term exposure to iAs through the consumption of these bivalves. The corresponding carcinogenic risk was 4.8 × 10^−5^, making the risk of developing cancer approximately 1 person out of every 20,000 inhabitants; this result is within the recommended reference interval (10^−4^–10^−6^) [58].

The speciation of iAs could be considered a limitation of this study; therefore, estimates were made assuming that the iAs/tAs ratio had different ratios and the associated health risks were calculated (Table 3). When high values, such as 30% and 50%, are assumed, the THQ risk remains well below 1, indicating that mussel consumption is not harmful to human health. However, when 30% is assumed, the acceptable CR is slightly exceeded (from 10^−4^ to 10^−6^), thus increasing the average lifetime risk of developing cancer. Considering the low probability of obtaining a global sample of mussels with an average iAs/tAs ratio equal to or greater than 30% [24,25,26,27,28,29,30,31], it is concluded that the mussels presented in this study remain safe for consumption.

**Table 3:** Health risks associated with the assumption of different iAs/tAs ratios in *Mytilus chilensis*.

iAs/tAs Ratio (%)	EDI iAs (μg/kg bw/day)	THQ	CR
5	0.016	0.053	2.4 × 10^−5^
10	0.032	0.107	4.8 × 10^−5^
20	0.064	0.213	9.6 × 10^−5^
30	0.096	0.320	1.4 × 10^−4^
50	0.160	0.533	2.4 × 10^−4^

### 3.3. Lead (Pb) in Specimens of M. chilensis by Study Area

**Figure 4:**
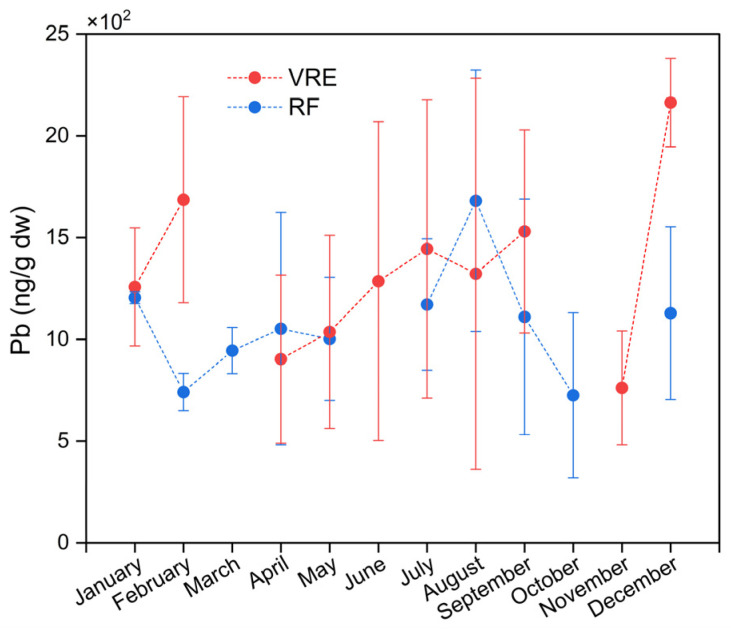
Lead concentrations in specimens of *M. chilensis* from the Valdivia River Estuary vs. Reloncaví Fjord according to the month of sampling.

The analysis of Pb concentrations in the samples of *M. chilensis* also showed the existence of differences in the areas, points, and months of sampling. Figure 4 represents the concentrations of Pb in both study areas by months of sampling. The global average of Pb in the samples collected from the VRE (1332 ± 664 ng/g) was higher than those from the RF (1072 ± 469 ng/g). In particular, the samples collected from the VRE in December (2163 ± 218 ng/g) (late spring/early summer) had the highest concentration; meanwhile, the lowest was obtained from those collected in November (762 ± 279 ng/g).

These results are from a study performed on the Algerian west coast with specimens of *M. galloprovincialis*, where the highest concentration of Pb was also detected in the samples collected during the summer from the port of Oran (15,470 ± 500 ng/g) [67]. This could be related to an increase in the urban population during that time of year, which may result in higher urban wastewater discharges. Additionally, this increase in Pb bioaccumulation by the mussels could also be due to an increase in the temperature during December [86].

Comparisons of the sampling points in the VRE revealed that the concentration of Pb in the samples collected from all three points was statistically similar (Figure 3). In the RF, the samples collected in August (which corresponds with the austral winter) had the highest Pb concentration (1681 ± 642 ng/g), while those collected in February (741 ± 92 ng/g) and October (725 ± 405 ng/g) had the lowest (Figure 4).

These results coincide with those obtained in France, where higher concentrations of Pb were reported during the winter, with an annual range of 1400–1700 ng/g [77]. In the previously mentioned study performed in Algeria, they also observed the highest concentration of Pb (5660 ± 100 ng/g) in the samples from the port of Hadjaj during the winter [67]. Similarly, Dresser Ort had the highest concentration of Pb (1064 ng/g) during the winter [78]. Other studies on trace elements in *Lucina pectinata* in Brazil also found that the samples with the highest concentration of Pb (15,100 ± 8400 ng/g) were collected during the rainy season, which coincides with the climatic conditions of the austral winter [77]. The increase in the metal concentrations of samples during the winter period could be influenced by anthropogenic activities, with industrial, agricultural, and domestic effluents entering the aquatic environment due to soil drainage during the rainy season [78].

When comparing the results obtained from the sampling points in the RF (Figure 3), it was observed that the samples collected from point VII had the highest concentration, with an average of 1281 ± 460 ng/g, while those collected from the point VI had the lowest concentration (784 ± 354 ng/g). Comparisons of the concentrations of Pb found in the samples, based on their wet weight (w.w.), revealed that they remained below the maximum limit for Pb in bivalve mollusks of 2000 ng/g w.w., which was established by the RSA for ‘canned fish and shellfish, fresh chilled and frozen fish and shellfish’, and that of 1500 ng/g w.w., established by the CONTAM Panel for the same [17,87].

The corresponding EDI for Pb in the analyzed samples was 0.01 µg/(kg bw day) which is lower than the values of 0.06 µg/(kg bw day) found in China [82], 0.16 µg/(kg bw day) in Spain [60], 0.17 µg/(kg bw day) in Argentina [66], 1 µg/(kg bw day) in Indonesia [84], 8 µg/(kg bw day) in Korea [88] and 149 µg/(kg bw day) in Turkey [62].

The corresponding THQ value was 0.00; at a value ˂ 1, the Chilean population should not experience any adverse health risks associated with long-term exposure to Pb from the consumption of *M. chilensis*. The corresponding carcinogenic risk was 4.3 × 10^−6^, making the risk of developing cancer approximately five persons out of every 1 million inhabitants, a result that is well within the recommended reference interval (10^−4^–10^−6^) [58].

### 3.4. Cadmium (Cd) in Specimens of M. chilensis by Study Area

**Figure 5:**
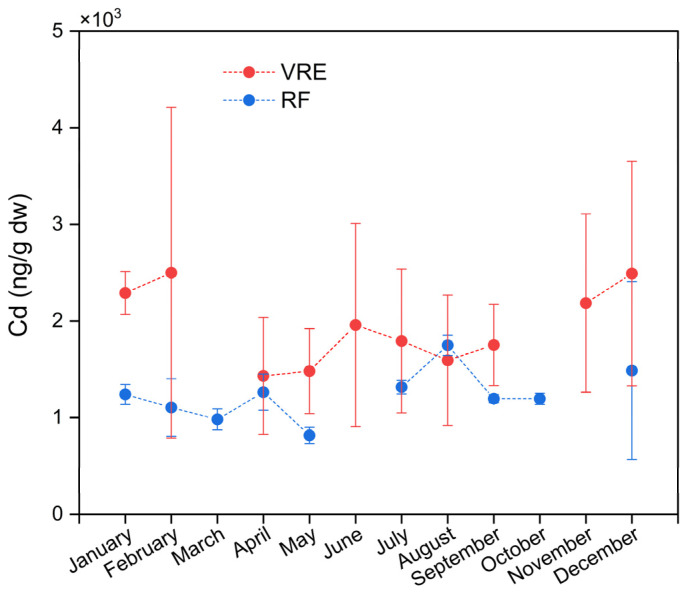
Cadmium concentrations in *M. chilensis* specimens from the Valdivia River Estuary vs. Reloncaví Fjord according to the month of sampling. Dashed lines represent months not sampled due to climatic difficulties.

Significant differences were found between the concentrations of Cd in the areas, points, and months of sampling. Samples collected from the VRE had a higher concentration (1954 ± 944 ng/g) than those from the RF (1235 ± 396 ng/g). In the VRE, the highest concentration was observed in the samples collected in February (2499 ± 1712 ng/g) and December (2491 ± 1162 ng/g). The lowest concentration was observed in the samples collected in May (1481 ± 439 ng/g) and April (1432 ± 606 ng/g) (Figure 5).

Focusing on the VRE sampling points, results in the VRE, and comparisons according to the sampling points revealed significant differences between all points (Figure 3). Samples collected from point I with an average of 2932 ± 890 ng/g were higher than those from point II (1107 ± 422 ng/g) and III (1921 ± 346 ng/g); those from point II had the lowest concentration.

In the study in Algeria, the highest Cd concentration (740 ± 70 ng/g) was recorded during the spring in the ports of Ain Defla and Hadjaj [67]. Meanwhile, in Chile, changes relating to the concentrations of Cd in the soft tissue and digestive gland in specimens of *M. chilensis* for one year (June 2014–June 2015) were reported at different ranges of depth in the Caucahue Channel, Yal Bay and Piti Palena Fjord [89]. In the Piti Palena Fjord, they reported a 34% increase in the concentration of Cd (from 4100 to 5500 ng/g) in the digestive glands of the samples collected during the spring. They attributed this increase to the chemical speciation of digestible Cd, which was available because of the nutritional characteristics of the spring seston. The digestible cadmium would then associate itself with peptides such as metallothioneins in the tissues of the mussels. In the Piti Palena fjord, they also observed a 17% decrease in the concentration of Cd in the digestive glands of the samples collected during the fall of 2015 (from 7500 to 6200 ng/g), which they attributed to the presence of indigestible Cd compounds produced by anoxic environments, an overload of organic matter and slow reduction–oxidation reactions. On the other hand, a study of heavy metals in specimens of *Bracchidontes rodriguezii*, collected from the Bahía Blanca Estuary, Argentina, reported lower concentrations of Cd during the winter [90].

In the RF, the samples collected in August (1749 ± 105 ng/g) had the highest average concentration, while those collected in May (817 ± 85 ng/g) had the lowest. In the fall, the concentration decreased from 1264 ± 187 ng/g in April to 817 ± 85 ng/g in May. Meanwhile, during the winter, it increased from 1316 ± 70 ng/g in July to 1749 ± 105 ng/g in August. In the spring, the samples collected in September and October presented statistically similar concentrations of 1196 ± 42 and 1196 ± 58 ng/g, respectively. Meanwhile, the concentration of those collected in December showed an increase (1487 ± 919 ng/g) (Figure 5).

The results recorded in the RF during August are similar to those of Blanc et al., who recorded a concentration of 1800 ng/g in the soft tissue of the samples during the winter of 2015 [81]. Coincidentally, Knopf et al. also observed the highest concentration of Cd (1226 ng/g) in samples collected from the Darsser Ort site during the winter season [78]. A study on the seasonal variations of heavy metals in *M. galloprovincialis* from the coast of Cala Iris (northern Morocco) found that the concentration of Cd was significantly higher during the winter [9]. They attributed this increase to the fact that mussels tend to bioaccumulate more heavy metals in the cold season, which coincides with their reproductive cycle that takes place in late winter–early spring. It was also indicated that the higher concentrations of Cd (820 ± 80 ng/g^)^ in the samples from the port of Oran during winter were due to changes in the weight of *M. galloprovincialis* during their reproductive cycle [67].

One study reported that in the late winter, during a period of abundant rainfall, mussels living in the northwestern Mediterranean accumulated a maximum concentration of trace metals [83]. This may have been so because those metals can enter and contaminate marine waters from tributaries, leaving them available to be absorbed and accumulated by mussels. Studies performed on heavy metals in specimens of *Lucina pectinata* in Brazil [77] and in *Perna viridis* in the Philippines [84] showed that the highest concentrations of Cd in their respective studies came from the samples collected during the rainy seasons in both countries. These differences are due to the reduction in the tissue mass of the mussels due to a lack of food during the cold season; therefore, the concentrations of metals expressed about the body weight of the mussels would be higher.

The analysis of the sampling points in the RF (Figure 3) also evidenced the existence of significant differences between them. The maximum concentration of Cd was obtained from the samples collected from point IV: 1330 ± 538 ng/g; meanwhile, those from points VI and VII had the lowest concentrations, with 1102 ± 258 ng/g and 1260 ± 297 ng/g.

With regard to the national legislation, the RSA still lacks data regarding the maximum allowable limits for Cd in bivalve mollusks; however, the CONTAM Panel and JECFA have established maximum limits of 1000 ng/g w.w. and 2000 ng/g w.w., respectively [17]. Comparisons of the mean concentrations of Cd obtained from all the analyzed samples on a wet-weight basis revealed that none of them exceeded the maximum limits established by the aforementioned legislation.

The corresponding EDI in the analyzed samples was 0.01 µg/(kg bw day), results that are much lower than the values of 0.07 µg/(kg bw day) found in Malaysia [91], 0.21 µg/(kg bw day) in Spain [60], 0.30 µg/(kg bw day) in Argentina [66], 0.56 µg/(kg bw day) in China [82], 2.5 µg/(kg bw day) in Indonesia [84] and 22 µg/(kg bw day) in Turkey [62].

The quantification of the risks associated with human health yielded a THQ of 0.01. Being <1, the population should not experience any health hazards associated with long-term exposure to Cd due to the consumption of *M. chilensis*. The CR of 6.9 × 10^−5^ means that approximately seven people out of every 100,000 inhabitants are likely to develop cancer from Cd ingestion.

The cumulative non-carcinogenic risk was evaluated by adding the Target Hazard Quotient (THQ) of iAs (0.02), Pb (0.00), and Cd (0.01), and allowed for the calculation of the Total Target Hazard Quotient (TTHQ). It was found that the TTHQ was 0.03 and it being <1 indicates that exposure to those metals through the consumption of specimens of *M. chilensis* from the south of the country poses no potential health risks to the Chilean population.

### 3.5. Environmental and Anthropogenic Factors

In general, the elevated levels of the studied metals in the samples collected from the VRE in the months of November–December (except for Pb in November) can be attributed to the fact that it is an environmental setting that has a particular lake-like structure. The rivers in the upper areas firstly flow into the lakes then they flow into the estuary. Subsequently, when the snowmelt begins around November, the lakes begin to release excess water into the estuary, along with metals and other suspended matter they may contain. This is also coupled with the contributions from the tributaries in the lower areas that also flow into the estuary. The Chilean Patagonia is characterized by continuous wet conditions throughout the year, subjected to abundant precipitation during the winter (June to August) and meltwater during spring/summer (September to January) [3]. Terrestrial runoff, freshwater inputs, and suspended particles from the sediments at the bottom of the water can also enrich the concentrations of toxic compounds in the aquatic environment [62].

In recent years, Valdivia’s estuarine ecosystem has been affected by an increasing rate of anthropogenic alterations, especially related to contamination by sewage, industrial and domestic waste, and agricultural and livestock activity [55]. In the VRE, the environmental concentration of heavy metals has increased due to the intensification of urbanization and other activities such as forestry terminals, fishmeal plants, commercial shipping, artisanal fishing, salmon farming, and tourism. In addition, effluents from industry, agriculture, forestry, and urban sources in the city of Valdivia are discharged into the rivers and constitute an important source of contamination and deterioration of the quality of the water [92].

On the other hand, the higher concentrations of the studied metals observed in the RF in August could be attributed to the increased flow of the rivers Petrohué, Puelo, and Cochamó during the winter because of increased precipitation. These rivers flow directly into the Reloncaví Fjord, and their discharges can reach a maximum between July and September, with a flow intensity reaching up to ~1500 m^3^/s [93]. This higher water flow can drag more metals directly into the fjord, increasing their concentration in that aquatic environment.

Seasonal changes in the concentration of various metals in mussel tissues could be the result of a combination of other factors directly correlated to the water temperature, pH, conductivity, and salinity, along with their ability to absorb or excrete contaminants [68,94]. However, no correlation was observed between the environmental parameters of the water registered in the RF and the VRE and the concentrations of the studied metals in this study. The main sources of contaminants for bivalves are dissolved chemicals in the water and contaminated food particles such as phytoplankton, particles, and microorganisms from seston and microplankton. They are filter feeders due to their sessile nature and can filter between 0.2 and 5 L of water per hour to meet their respiratory and nutritional needs [94,95]. It is possible that the high levels of heavy metals in the mussels’ tissues detected during warmer months in the VRE could be related to an increase in food particles in those months, as they could act as regular heavy metal carriers for mussels [90].

Generally, the overall risk of environmental contamination when heavy metals enter the food chain depends mainly on the physicochemical properties of the contaminating substance, such as (a) the amount of heavy metals present in water and sediments, (b) the forms that can be solubilized and (c) the heavy metals that can be maintained in an exchangeable form on the surface of suspended particles [96]. The concentrations of contaminants vary seasonally in the *Mytilus* spp., as their bioaccumulation of heavy metals depends not only on the environmental concentrations of the same and the availability of food, but also on several biological factors such as the mussel’s age, size, sex, reproductive cycle, and metabolism [97].

### 3.6. Bioaccessibility of Total Arsenic, Lead, and Cadmium in Specimens of M. chilensis

#### 3.6.1. Bioaccessibility of Total Arsenic (tAs)

**Figure 6:**
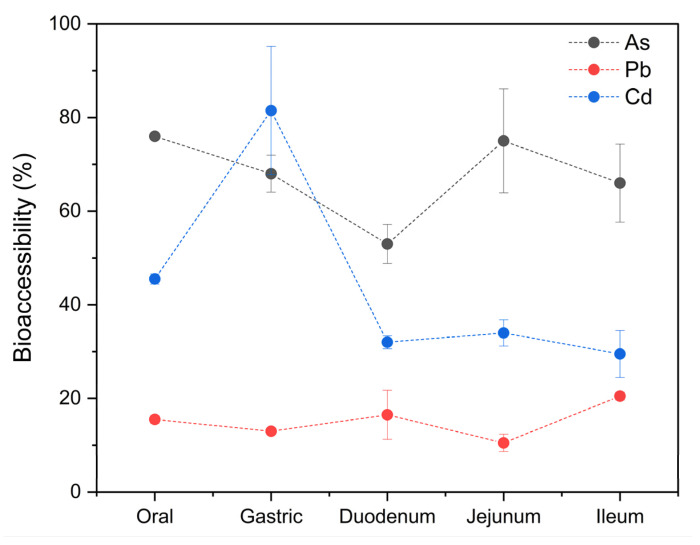
Bioaccessibility of tAs, Pb, and Cd in specimens of *M. chilensis*.

The mean tAs concentration obtained after the mineralization was 2053 ± 40 ng/g wet weight (w.w.), with a range of 2025–2081 ng/g w.w. After the in vitro gastrointestinal digestion assay, the concentration of the bioaccessible fraction was 68 ± 10% (1388 ± 205 ng/g w.w.), ranging from 50 to 83% (1036–1701 ng/g w.w.). Statistical differences were observed between the bioaccessible percentages of tAs and the different stages of digestion. In Figure 6, a high bioaccessibility can be observed in the oral stage (76%), with a slight and constant decrease in the gastric (68%) and duodenal (53%) stages, followed by an increase in the jejunum (75%), and ending with a slight decrease in the ileum (66%).

These general results coincide with those of Leufroy [98], who found a bioaccessibility of 84% for tAs in samples of mixed mussels, and Torres-Escribano et al. [99], who found a bioaccessibility ranging from 47 to 72% for tAs about food materials of *Fucus* sp. and lobster hepatopancreas. Bioaccessibility values of above 50% indicate that the majority of the arsenic in the studied matrix is in its organic form, in compounds such as arsenobetaine, arsenocoline, and arsenosugars [99,100,101,102]. The high bioaccessibility of tAs in general and in the oral stage may be due to the solubility of arsenosugars and other compounds of organic arsenic in water [99]. This theory is supported by the results of FAO [5], who observed that 40% of the arsenobetaine in the reference food materials (*Fucus* sp. and lobster hepatopancreas) in their study was solubilized by saliva. The findings from studies performed by Leufroy [103] and Calatayud [104] demonstrated that arsenic released by saliva accounts for at least half of the bioaccessible arsenic in shellfish samples.

The increased bioaccessibility of tAs in the jejunum could have resulted from the increased release of arsenobetaine in the oral stage [104]. The bioaccessibility of arsenobetaine is higher in the intestine than in the stomach for raw and cooked seafood [101]. They attributed that increase to the release of arsenobetaine from the structures in shellfish, as it is a process that requires more time and enzymes. On the other hand, arsenic is known to bind primarily to metallothionein-like proteins (MTLPs) in mussels and fish [96]. In humans, proteins can be hydrolyzed into polypeptides by proteases in the gastric juice and are then broken down into oligopeptides and amino acids by trypsin in the intestine; similarly, arsenic in food can be gradually released from protein along the gastrointestinal tract [55,105].

#### 3.6.2. Bioaccessibility of Lead (Pb)

The mean Pb concentration obtained after the mineralization was 4762 ± 129 ng/g w.w., with a range of 4670–4853 ng/g w.w. The concentration of the bioaccessible fraction was 15 ± 4% (726 ± 187 ng/g w.w.), ranging from 9 to 21% (445–992 ng/g w.w.). These results are lower than those reported in *M. galloprovincialis* (50.1–78.4%) [44] and the % in oysters (28.3–51.4%) [106]. In the case of Pb, the percentages of bioaccessibility were relatively constant throughout the digestion process; however, there were significant differences between stages. It started in the oral stage with 15.5%, dropped to 13% in the gastric stage, and rose to 16.5% in the duodenum, after which it dropped again to 10.5% in the jejunum, increasing to 20.5% in the ileum (Figure 6). Lead is present in numerous types of cells in the tissues of mollusks. It is distributed in the lysosomes and cytosol, which are the main intracellular structures responsible for its storage in the gills and the digestive tract [107]. Therefore, its bioaccessibility is limited due to this storage mechanism [106].

#### 3.6.3. Bioaccessibility of Cadmium (Cd)

The mean Cd concentration obtained after the mineralization was 760 ± 3 ng/g w.w., with a range of 762–758 ng/g w.w. The concentration of the bioaccessible fraction was 45 ± 21% (338 ± 158 ng/g w.w.), ranging from 26 to 91% (200–691 ng/g w.w.). Similar values have been reported for oysters (33.8–59.2%) [106], eastern oysters and shrimp (45%) [48], and *M. galloprovincialis* (55.20–88.62%) [44]. Significant differences were also observed between the different stages of digestion and the bioaccessible percentages of Cd found in the analyzed samples (Figure 6). The bioaccessibility of this metal reached its highest point in the gastric stage (81.5%), after starting in the mouth at 45.5%. Another study also found that Cd was most bioaccessible in the gastric stage [48]. This is because in mollusks, Cd is tightly bound to thiol proteins such as metallothionein, and this soluble protein is easily degraded in the digestive tract of consumers, releasing the bound Cd into its matrix [43]. Subsequently, the bioaccessibility drastically reduced in the duodenum (32%), after which it steadily decreased to 29.5% in the ileum. These results coincide with findings published by Waisberg et al. [100], who also found a progressive decrease in the bioaccessibility of Cd in the intestinal phases in their study and stated that it can be explained by the fact that Cd decreases proportionally as pH increases. It is also possible that while the pH increases, the metallothioneins are reactivated and sequester the Cd that was previously released in the gastric stage.

In general, tAs were found to have a higher mean percentage of bioaccessibility than the other two metals. The difference among them could be based on their respective natures, the species studied, and the sampling area [44,45,48].

## 4. Conclusions

The results show that the areas, points, and months of sampling led to differences in the mean concentrations of each of the metals analyzed. No correlation was observed between the environmental parameters of the water (temperature, salinity, conductivity, and pH) in both study areas and the mean concentrations of the contaminants analyzed in this study. The absence of a direct correlation between environmental parameters and metal concentrations suggests that the bioaccumulation mechanisms in *M. chilensis* are complex and dependent on multiple factors.

The concentrations of tAs, Pb, and Cd in all samples were below the maximum limits established by the national and international legislations.

The tAs proved to be the most bioaccessible of the three contaminants studied. The non-carcinogenic risk was evaluated by the calculation of the Total Target Hazard Quotient (TTHQ), which was performed by adding the Target Hazard Quotients of iAs (0.02), Pb (0.00), and Cd (0.01). This summation produced a value of 0.03, which (because it’s <1) indicates that there are no potential health risks posed to the Chilean population by exposure to these metals through the consumption of *M. chilensis*.

The quantification of the carcinogenic risk (CR) associated with the ingestion of iAs, Pb, and Cd did not exceed the safety levels, which also suggests that exposure to these metals associated with the consumption of *M. chilensis* in Chile is not dangerous for consumers. However, it is important to remember that only this food was evaluated in this study.

The constant monitoring of the concentrations of heavy metals in these populations of bivalves is recommended, given that they are a very important resource at the national level in terms of production, consumption, and export. It is recommended that future investigations are carried out to address the speciation of tAs, the seasonal variability across years, and the impact of environmental conditions on natural mussel banks, including their fertility and reproduction. These aspects were not considered in the present study, but their analysis will allow for a more comprehensive understanding of the dynamics of these ecosystems. It is essential that the Ministry of Health issue and keep updated regulations for the maximum allowable limits for all heavy metals in this food matrix, as well as in other matrices, guaranteeing their innocuity along with the health and safety of consumers.

## Data Availability

All data and materials are available upon request from the corresponding author. The data are not publicly available due to ongoing research using part of the data.

## References

[1] Lagos L., Uriarte I., Yany G. (2012). Evaluacion Del Potencial Reproductivo Del Chorito (*Mytilus chilensis*) de Dos Poblaciones Naturales Sometidas a Diferentes Temperaturas de Acondicionamiento. Lat. Am. J. Aquat. Res..

[2] Flores C.A.M., Gomez M.A.D., Muñoz C.B.A., Pérez L.E.C., Arribas S.L.M., Opazo M.P.A., Huaquin E.J.E.N. (2015). Spatial Distribution Pattern of *Mytilus chilensis* Beds in the Reloncaví Fjord: Hypothesis on Associated Processes. Rev. Chil. Hist. Nat..

[3] Yevenes M.A., Lagos N.A., Farías L., Vargas C.A. (2019). Greenhouse Gases, Nutrients and the Carbonate System in the Reloncaví Fjord (Northern Chilean Patagonia): Implications on Aquaculture of the Mussel, *Mytilus chilensis*, during an Episodic Volcanic Eruption. Sci. Total Environ..

[4] Subpesca (2021). Informe Sectorial de Pesca y Acuicultura.

[5] FAO Information Group, FAO (2000). The State of World Fisheries and Aquaculture.

[6] Bastías J.M., Moreno J., Pia C., Reyes J., Quevedo R., Muñoz O. (2015). Effect of Ohmic Heating on Texture, Microbial Load, and Cadmium and Lead Content of Chilean Blue Mussel (*Mytilus chilensis*). Innov. Food Sci. Emerg. Technol..

[7] Santibáñez P., Romalde J., Maldonado J., Fuentes D., Figueroa J. (2022). First Characterization of the Gut Microbiome Associated with *Mytilus chilensis* Collected at a Mussel Farm and from a Natural Environment in Chile. Aquaculture.

[8] Hervé-Fernández P., Houlbrèque F., Boisson F., Mulsow S., Teyssié J.-L., Oberhaënsli F., Azemard S., Jeffree R. (2010). Cadmium Bioaccumulation and Retention Kinetics in the Chilean Blue Mussel *Mytilus chilensis*: Seawater and Food Exposure Pathways. Aquat. Toxicol..

[9] Silva dos Santos F., Neves R.A.F., Crapez M.A.C., Teixeira V.L., Krepsky N. (2022). How Does the Brown Mussel Perna Perna Respond to Environmental Pollution? A Review on Pollution Biomarkers. J. Environ. Sci..

[10] Fiori C.d.S., Rodrigues A.P.d.C., Vieira T.C., Sabadini-Santos E., Bidone E.D. (2018). An Alternative Approach to Bioaccumulation Assessment of Methyl-Hg, Total-Hg, Cd, Pb, Zn in Bivalve Anomalocardia Brasiliana from Rio de Janeiro Bays. Mar. Pollut. Bull..

[11] Ozkan D., Dagdeviren M., Katalay S., Guner A., Yavaşoğlu N.Ü.K. (2017). Multi-Biomarker Responses After Exposure to Pollution in the Mediterranean Mussels (*Mytilus galloprovincialis* L.) in the Aegean Coast of Turkey. Bull. Environ. Contam. Toxicol..

[12] Lozano G., Herraiz E., Hardisson A., Gutiérrez A.J., González-Weller D., Rubio C. (2010). Heavy and Trace Metal Concentrations in Three Rockpool Shrimp Species (*Palaemon elegans*, *Palaemon adspersus* and *Palaemon serratus*) from Tenerife (Canary Islands). Environ. Monit. Assess..

[13] Squadrone S., Brizio P., Stella C., Prearo M., Pastorino P., Serracca L., Ercolini C., Abete M.C. (2016). Presence of Trace Metals in Aquaculture Marine Ecosystems of the Northwestern Mediterranean Sea (Italy). Environ. Pollut..

[14] Figueira E., Lima A., Branco D., Quintino V., Rodrigues A.M., Freitas R. (2011). Health Concerns of Consuming Cockles (*Cerastoderma edule* L.) from a Low Contaminated Coastal System. Environ. Int..

[15] Hossen F., Hamdan S., Rahman R. (2015). Review on the Risk Assessment of Heavy Metals in Malaysian Clams. Sci. World J..

[16] Orisakwe O.E., Nduka J.K., Amadi C.N., Dike D.O., Bede O. (2012). Heavy Metals Health Risk Assessment for Population via Consumption of Food Crops and Fruits in Owerri, South Eastern, Nigeria. Chem. Cent. J..

[17] European Commission E. (2006). Commission Regulation (EC) No 1881/2006 of 19 December 2006 Setting Maximum Levels for Certain Contaminants in Foodstuffs. Off. J. Eur. Union.

[18] Min H.-G., Kim M.-S., Kim J.-G. (2021). Effect of Soil Characteristics on Arsenic Accumulation in Phytolith of Gramineae (*Phragmites japonica*) and Fern (*Thelypteris palustris*) Near the Gilgok Gold Mine. Sustainability.

[19] ATSDR ATSDR Substance Priority List Available. https://www.atsdr.cdc.gov/programs/substance-priority-list.html?CDC_AAref_Val=https://www.atsdr.cdc.gov/spl/index.html.

[20] Turner M.C., Cogliano V., Guyton K., Madia F., Straif K., Ward E.M., Schubauer-Berigan M.K. (2023). Research Recommendations for Selected IARC-Classified Agents: Impact and Lessons Learned. Environ. Health Perspect..

[21] González L., Muñoz-Fariña O., Fernández-Guerrero Y., Roman-Benn A., Bastias-Montes J.M., Quevedo-León R., Ravanal M.C. (2024). Arsenic, Lead and Cadmium Concentration in Food and Estimated Daily Intake in the Cuban Population and the Health Risks Using a Total Diet Study. J. Environ. Sci. Health Part B.

[22] Muñoz O., Zamorano P., Garcia O., Bastías J.M. (2017). Arsenic, Cadmium, Mercury, Sodium, and Potassium Concentrations in Common Foods and Estimated Daily Intake of the Population in Valdivia (Chile) Using a Total Diet Study. Food Chem. Toxicol..

[23] WHO (1996). Chapter 15 Arsenic.

[24] Sloth J.J., Julshamn K. (2008). Survey of Total and Inorganic Arsenic Content in Blue Mussels (*Mytilus edulis* L.) from Norwegian Fiords: Revelation of Unusual High Levels of Inorganic Arsenic. J. Agric. Food Chem..

[25] Vélez D., Montoro R. (2001). Inorganic Arsenic in Foods: Current Overview and Future Challenges. Recent Res. Dev. Agric. Food Chem..

[26] Buchet J.P., Pauwels J., Lauwerys R. (1994). Assessment of Exposure to Inorganic Arsenic Following Ingestion of Marine Organisms by Volunteers. Environ. Res..

[27] Gomez-Delgado A.I., Tibon J., Silva M.S., Lundebye A.-K., Agüera A., Rasinger J.D., Strohmeier T., Sele V. (2023). Seasonal Variations in Mercury, Cadmium, Lead and Arsenic Species in Norwegian Blue Mussels (*Mytilus edulis* L.)—Assessing the Influence of Biological and Environmental Factors. J. Trace Elem. Med. Biol..

[28] Kucuksezgin F., Gonul L.T., Tasel D. (2014). Total and Inorganic Arsenic Levels in Some Marine Organisms from Izmir Bay (Eastern Aegean Sea): A Risk Assessment. Chemosphere.

[29] Ruangwises S., Ruangwises N. (2011). Concentrations of Total and Inorganic Arsenic in Fresh Fish, Mollusks, and Crustaceans from the Gulf of Thailand. J. Food Prot..

[30] Argese E., Bettiol C., Rigo C., Bertini S., Colomban S., Ghetti P.F. (2005). Distribution of Arsenic Compounds in *Mytilus galloprovincialis* of the Venice Lagoon (Italy). Sci. Total Environ..

[31] Muñoz O., Devesa V., Suñer M.A., Vélez D., Montoro R., Urieta I., Macho M.L., Jalón M. (2000). Total and Inorganic Arsenic in Fresh and Processed Fish Products. J. Agric. Food Chem..

[32] Gidlow D.A. (2015). Lead Toxicity. Occup. Med..

[33] Yabe J., Nakayama S.M.M., Ikenaka Y., Yohannes Y.B., Bortey-Sam N., Kabalo A.N., Ntapisha J., Mizukawa H., Umemura T., Ishizuka M. (2018). Lead and Cadmium Excretion in Feces and Urine of Children from Polluted Townships near a Lead-Zinc Mine in Kabwe, Zambia. Chemosphere.

[34] Rahbar N., Nazari Z. (2004). Level of Lead and Cadmium in Peanut. Feyz Med. Sci. J..

[35] Patrick L. (2006). Lead Toxicity, A Review of the Literature. Part I: Exposure, Evaluation, and Treatment. Altern. Med. Rev..

[36] Domeneh B.H., Tavakoli N., Jafari N. (2014). Blood Lead Level in Opium Dependents and Its Association with Anemia: A Cross-Sectional Study from the Capital of Iran. J. Res. Med. Sci..

[37] Maxwell L. (2020). Absorption, Distribution, and Excretion in Complex Organisms. An Introduction to Interdisciplinary Toxicology.

[38] EFSA (2010). Scientific Opinion on Lead in Food. EFSA J..

[39] ATSDR (2021). Lead Toxicity Case Studies in Environmental Medicine.

[40] Lin G., Chen T., Pan Y., Yang Z., Li L., Yong K., Wang X., Wang J., Chen Y., Jiang W. (2020). Biodistribution and Acute Toxicity of Cadmium-Free Quantum Dots with Different Surface Functional Groups in Mice Following Intratracheal Inhalation. Nanotheranostics.

[41] Chen J., Kang D., Yan Z., Shen Q., Lou Y., Li Y., Kong A., Pan B., Huang C. (2019). Tissue Distribution of Tetrabromobisphenol A and Cadmium in Mixture Inhalation Exposure. Toxicol. Ind. Health.

[42] EFSA (2012). Cadmium Dietary Exposure in the European Population. EFSA J..

[43] Amiard J.-C., Amiard-Triquet C., Charbonnier L., Mesnil A., Rainbow P.S., Wang W.-X. (2008). Bioaccessibility of Essential and Non-Essential Metals in Commercial Shellfish from Western Europe and Asia. Food Chem. Toxicol..

[44] Gedik K. (2018). Bioaccessibility of Cd, Cr, Cu, Mn, Ni, Pb, and Zn in Mediterranean Mussel (*Mytilus galloprovincialis* Lamarck, 1819) along the Southeastern Black Sea Coast. Hum. Ecol. Risk Assess. Int. J..

[45] He M., Wang W.-X. (2013). Bioaccessibility of 12 Trace Elements in Marine Molluscs. Food Chem. Toxicol..

[46] Lucas-González R., Viuda-Martos M., Pérez-Alvarez J.A., Fernández-López J. (2018). In Vitro Digestion Models Suitable for Foods: Opportunities for New Fields of Application and Challenges. Food Res. Int..

[47] Versantvoort C.H.M., Oomen A.G., Van de Kamp E., Rompelberg C.J.M., Sips A.J.A.M. (2005). Applicability of an in Vitro Digestion Model in Assessing the Bioaccessibility of Mycotoxins from Food. Food Chem. Toxicol..

[48] Wang C., Duan H.-Y., Teng J.-W. (2014). Assessment of Microwave Cooking on the Bioaccessibility of Cadmium from Various Food Matrices Using an In Vitro Digestion Model. Biol. Trace Elem. Res..

[49] Wickham M., Faulks R., Mills C. (2009). In Vitro Digestion Methods for Assessing the Effect of Food Structure on Allergen Breakdown. Mol. Nutr. Food Res..

[50] Jones D., Caballero S., Davidov-Pardo G. (2019). Bioavailability of Nanotechnology-Based Bioactives and Nutraceuticals. Adv. Food Nutr. Res..

[51] Navarro-Alarcon M., Cabrera-Vique C. (2008). Selenium in Food and the Human Body: A Review. Sci. Total Environ..

[52] Azizur Rahman M., Hasegawa H., Peter Lim R. (2012). Bioaccumulation, Biotransformation and Trophic Transfer of Arsenic in the Aquatic Food Chain. Environ. Res..

[53] AOAC (2000). Official Methods of Analysis. Determination of Lead, Cadmium, and Minerals in Food by Atomic Absorption Spectrophotometry.

[54] Ah-Hen K.S., Mathias-Rettig K., Gómez-Pérez L.S., Riquelme-Asenjo G., Lemus-Mondaca R., Muñoz-Fariña O. (2018). Bioaccessibility of Bioactive Compounds and Antioxidant Activity in Murta (*Ugni molinae* T.) Berries Juices. J. Food Meas. Charact..

[55] Lyu R., Gao Z., Li D., Yang Z., Zhang T. (2020). Bioaccessibility of Arsenic from Gastropod along the Xiangjiang River: Assessing Human Health Risks Using an in Vitro Digestion Model. Ecotoxicol. Environ. Saf..

[56] Saha N., Mollah M.Z.I., Alam M.F., Safiur Rahman M. (2016). Seasonal Investigation of Heavy Metals in Marine Fishes Captured from the Bay of Bengal and the Implications for Human Health Risk Assessment. Food Control.

[57] Saha N., Zaman M.R. (2013). Evaluation of Possible Health Risks of Heavy Metals by Consumption of Foodstuffs Available in the Central Market of Rajshahi City, Bangladesh. Environ. Monit. Assess..

[58] USEPA Risk-Based Concentration. https://archive.epa.gov/region9/superfund/web/html/index-23.html.

[59] Novakov N.J., Kartalović B.D., Mihaljev Ž.A., Mastanjević K.M., Stojanac N.S., Habschied K.J. (2021). Heavy Metals and PAHs in Mussels on the Serbian Market and Consumer Exposure. Food Addit. Contam. Part B.

[60] Rodríguez-Hernández Á., Zumbado M., Henríquez-Hernández L.A., Boada L.D., Luzardo O.P. (2019). Dietary Intake of Essential, Toxic, and Potentially Toxic Elements from Mussels (*Mytilus* spp.) in the Spanish Population: A Nutritional Assessment. Nutrients.

[61] Joksimovic D., Stankovic S. (2012). The Trace Metals Accumulation in Marine Organisms of the Southeastern Adriatic Coast, Montenegro. J. Serbian Chem. Soc..

[62] Belivermiş M., Kılıç Ö., Çotuk Y. (2016). Assessment of Metal Concentrations in Indigenous and Caged Mussels (*Mytilus galloprovincialis*) on Entire Turkish Coastline. Chemosphere.

[63] Muñoz O., Cid H., Ah-Hen K., Bastías J.M. (2014). Cambios En Los Contenidos de Metales Pesados (Arsénico, Cadmio y Mercurio) En Productos Pesqueros Durante Los Procesos de Cocción. Agro Sur.

[64] Klarić S., Pavičić-Hamer D., Lucu Č. (2004). Seasonal Variations of Arsenic in Mussels *Mytilus galloprovincialis*. Helgol. Mar. Res..

[65] Velazquez Hernández D.M. (2005). Determinación de Metales Pesados en Biota (*Mytilus chilensis*) y Sedimentos de la Bahía de Corral, Provincia de Valdivia, X Región. Diploma Thesis.

[66] Conti M.E., Stripeikis J., Finoia M.G., Tudino M.B. (2011). Baseline Trace Metals in Bivalve Molluscs from the Beagle Channel, Patagonia (Argentina). Ecotoxicology.

[67] Rouane-Hacene O., Boutiba Z., Belhaouari B., Guibbolini-Sabatier M.E., Francour P., Risso-de Faverney C. (2015). Seasonal Assessment of Biological Indices, Bioaccumulation and Bioavailability of Heavy Metals in Mussels *Mytilus galloprovincialis* from Algerian West Coast, Applied to Environmental Monitoring. Oceanologia.

[68] Kamaruzzam B.Y., Zahir M.S.M., John B.A., Jalal K.C.A., Shahbudin S., Al-Barwani S.M., Goddard J.S. (2010). Bioaccumulation of Some Metals by Green Mussel Perna Viridis (Linnaeus 1758) from Pekan, Pahang, Malaysia. Int. J. Biol. Chem..

[69] Fatoki O.S., Okoro H.K., Adekola F.A., Ximba B.J., Snyman R.G. (2012). Bioaccumulation of Metals in Black Mussels (*Mytilus galloprovincialis*) in Cape Town Harbour, South Africa. Environmentalist.

[70] Lu G., Zhu A., Fang H., Dong Y., Wang W.-X. (2019). Establishing Baseline Trace Metals in Marine Bivalves in China and Worldwide: Meta-Analysis and Modeling Approach. Sci. Total Environ..

[71] Szefer P., Fowler S.W., Ikuta K., Osuna F.P., Ali A.A., Kim B.-S., Fernandes H.M., Belzunce M.-J., Guterstam B., Kunzendorf H. (2006). A Comparative Assessment of Heavy Metal Accumulation in Soft Parts and Byssus of Mussels from Subarctic, Temperate, Subtropical and Tropical Marine Environments. Environ. Pollut..

[72] Shulkin V.M., Presley B.J., Kavun V. (2003). Metal Concentrations in Mussel Crenomytilus Grayanus and Oyster Crassostrea Gigas in Relation to Contamination of Ambient Sediments. Environ. Int..

[73] Boisson F., Cotret O., Fowler S.W. (1998). Bioaccumulation and Retention of Lead in the Mussel *Mytilus galloprovincialis* Following Uptake from Seawater. Sci. Total Environ..

[74] Falcó G., Llobet J.M., Bocio A., Domingo J.L. (2006). Daily Intake of Arsenic, Cadmium, Mercury, and Lead by Consumption of Edible Marine Species. J. Agric. Food Chem..

[75] Fattorini D., Notti A., Di Mento R., Cicero A.M., Gabellini M., Russo A., Regoli F. (2008). Seasonal, Spatial and Inter-Annual Variations of Trace Metals in Mussels from the Adriatic Sea: A Regional Gradient for Arsenic and Implications for Monitoring the Impact of off-Shore Activities. Chemosphere.

[76] Esposito G., Mudadu A.G., Abete M.C., Pederiva S., Griglione A., Stella C., Ortu S., Bazzoni A.M., Meloni D., Squadrone S. (2021). Seasonal Accumulation of Trace Elements in Native Mediterranean Mussels (*Mytilus galloprovincialis* Lamarck, 1819) Collected in the Calich Lagoon (Sardinia, Italy). Environ. Sci. Pollut. Res..

[77] Devier M.-H., Augagneur S., Budzinski H., Le Menach K., Mora P., Narbonne J.-F., Garrigues P. (2005). One-Year Monitoring Survey of Organic Compounds (PAHs, PCBs, TBT), Heavy Metals and Biomarkers in Blue Mussels from the Arcachon Bay, France. J. Environ. Monit..

[78] Knopf B., Fliedner A., Radermacher G., Rüdel H., Paulus M., Pirntke U., Koschorreck J. (2020). Seasonal Variability in Metal and Metalloid Burdens of Mussels: Using Data from the German Environmental Specimen Bank to Evaluate Implications for Long-Term Mussel Monitoring Programs. Environ. Sci. Eur..

[79] Phillips D.J.H. (1976). The Common Mussel *Mytilus edulis* as an Indicator of Pollution by Zinc, Cadmium, Lead and Copper. I. Effects of Environmental Variables on Uptake of Metals. Mar. Biol..

[80] Vieira K.S., Delgado J.F., Lima L.S., Souza P.F., Crapez M.A.C., Correa T.R., Aguiar V.M.C., Baptista Neto J.A., Fonseca E.M. (2021). Human Health Risk Assessment Associated with the Consumption of Mussels (*Perna perna*) and Oysters (*Crassostrea rhizophorae*) Contaminated with Metals and Arsenic in the Estuarine Channel of Vitória Bay (ES), Southeast Brazil. Mar. Pollut. Bull..

[81] Nekhoroshkov P.S., Bezuidenhout J., Frontasyeva M.V., Zinicovscaia I.I., Yushin N.S., Vergel K.N., Petrik L. (2021). Trace Elements Risk Assessment for Consumption of Wild Mussels along South Africa Coastline. J. Food Compos. Anal..

[82] Liu Q., Liao Y., Shou L. (2018). Concentration and Potential Health Risk of Heavy Metals in Seafoods Collected from Sanmen Bay and Its Adjacent Areas, China. Mar. Pollut. Bull..

[83] Cano-Sancho G., Perelló G., Maulvault A.L., Marques A., Nadal M., Domingo J.L. (2015). Oral Bioaccessibility of Arsenic, Mercury and Methylmercury in Marine Species Commercialized in Catalonia (Spain) and Health Risks for the Consumers. Food Chem. Toxicol..

[84] Putri A.K., Barokah G.R., Andarwulan N. (2017). Human Health Risk Assessment of Heavy Metals Bioaccumulation In Fish and Mussels from Jakarta Bay. Squalen Bull. Mar. Fish. Postharvest Biotechnol..

[85] Bogdanović T., Ujević I., Sedak M., Listeš E., Šimat V., Petričević S., Poljak V. (2014). As, Cd, Hg and Pb in Four Edible Shellfish Species from Breeding and Harvesting Areas along the Eastern Adriatic Coast, Croatia. Food Chem..

[86] Gooddy D.C., Shand P., Kinniburgh D.G., Van Riemsdijk W.H. (1995). Field-based Partition Coefficients for Trace Elements in Soil Solutions. Eur. J. Soil. Sci..

[87] RSA (2021). Reglamento Sanitario de Los Alimentos-Decreto 977/1996.

[88] Mok J.S., Yoo H.D., Kim P.H., Yoon H.D., Park Y.C., Kim J.H., Kwon J.Y., Son K.T., Lee H.J., Ha K.S. (2014). Bioaccumulation of Heavy Metals in the Mussel *Mytilus galloprovincialis* in the Changseon Area, Korea, and Assessment of Potential Risk to Human Health. Fish. Aquat. Sci..

[89] Max Blanc J., Molinet C., Díaz P.A., Subiabre R., Salamanca M., Duemler J. (2019). Drastic Difference in Cadmium Concentration in Mussels (*Mytilus chilensis*) Observed between Seasons in Natural Bed and Aquaculture Systems in Chile. Environ. Monit. Assess..

[90] Buzzi N.S., Oliva A.L., Arias A.H., Marcovecchio J.E. (2017). Assessment of Trace Metal Accumulation in Native Mussels (*Brachidontes rodriguezii*) from a South American Temperate Estuary. Environ. Sci. Pollut. Res..

[91] Yap C.K., Cheng W.H., Karami A., Ismail A. (2016). Health Risk Assessments of Heavy Metal Exposure via Consumption of Marine Mussels Collected from Anthropogenic Sites. Sci. Total Environ..

[92] Silva C., Ferreira J.G., Bricker S.B., DelValls T.A., Martín-Díaz M.L., Yáñez E. (2011). Site Selection for Shellfish Aquaculture by Means of GIS and Farm-Scale Models, with an Emphasis on Data-Poor Environments. Aquaculture.

[93] Castillo M.I., Cifuentes U., Pizarro O., Djurfeldt L., Caceres M. (2016). Seasonal Hydrography and Surface Outflow in a Fjord with a Deep Sill: The Reloncaví Fjord, Chile. Ocean. Sci..

[94] Beyer J., Green N.W., Brooks S., Allan I.J., Ruus A., Gomes T., Bråte I.L.N., Schøyen M. (2017). Blue Mussels (*Mytilus edulis* spp.) as Sentinel Organisms in Coastal Pollution Monitoring: A Review. Mar. Environ. Res..

[95] Luoma S.N., Rainbow P.S. (2005). Why Is Metal Bioaccumulation So Variable? Biodynamics as a Unifying Concept. Environ. Sci. Technol..

[96] Astorga España M.S., Peña Méndez E.M., Lecaros Palma O., García Montelongo F.J. (1998). Heavy Metals in *Mytilus chilensis* from the Strait of Magallenes (Chile). Mar. Pollut. Bull..

[97] Mubiana V.K., Blust R. (2007). Effects of Temperature on Scope for Growth and Accumulation of Cd, Co, Cu and Pb by the Marine Bivalve *Mytilus edulis*. Mar. Environ. Res..

[98] Montojo U.M., Baldoza B.J.S., Cambia F.D., Benitez K.C.D., Perelonia K.B.S., Rivera A.T.F. (2021). Levels and Health Risk Assessment of Mercury, Cadmium, and Lead in Green Mussel (*Perna viridis*) and Oyster (*Crassostrea iredalei*) Harvested around Manila Bay, Philippines. Food Control.

[99] Torres-Escribano S., Denis S., Blanquet-Diot S., Calatayud M., Barrios L., Vélez D., Alric M., Montoro R. (2011). Comparison of a Static and a Dynamic in Vitro Model to Estimate the Bioaccessibility of As, Cd, Pb and Hg from Food Reference Materials Fucus sp. (IAEA-140/TM) and Lobster Hepatopancreas (TORT-2). Sci. Total Environ..

[100] Krishnakumar P.K., Qurban M.A., Stiboller M., Nachman K.E., Joydas T.V., Manikandan K.P., Mushir S.A., Francesconi K.A. (2016). Arsenic and Arsenic Species in Shellfish and Finfish from the Western Arabian Gulf and Consumer Health Risk Assessment. Sci. Total Environ..

[101] Fu Y., Yin N., Cai X., Du H., Wang P., Sultana M.S., Sun G., Cui Y. (2021). Arsenic Speciation and Bioaccessibility in Raw and Cooked Seafood: Influence of Seafood Species and Gut Microbiota. Environ. Pollut..

[102] Fattorini D., Alonso-Hernandez C.M., Diaz-Asencio M., Munoz-Caravaca A., Pannacciulli F.G., Tangherlini M., Regoli F. (2004). Chemical Speciation of Arsenic in Different Marine Organisms: Importance in Monitoring Studies. Mar. Environ. Res..

[103] Leufroy A., Noël L., Beauchemin D., Guérin T. (2012). Bioaccessibility of Total Arsenic and Arsenic Species in Seafood as Determined by a Continuous Online Leaching Method. Anal. Bioanal. Chem..

[104] Calatayud M., Xiong C., Du Laing G., Raber G., Francesconi K., van de Wiele T. (2018). Salivary and Gut Microbiomes Play a Significant Role in in Vitro Oral Bioaccessibility, Biotransformation, and Intestinal Absorption of Arsenic from Food. Environ. Sci. Technol..

[105] Goodman B.E. (2010). Insights into Digestion and Absorption of Major Nutrients in Humans. Adv. Physiol. Educ..

[106] Intawongse M., Sriraksa S., Dean J.R., Kongchana P. (2012). Estimation of Bioaccessibility of Heavy Metals in Oysters Using the Physiologically Based Extraction Test. Instrum. Sci. Technol..

[107] Marigómez I., Soto M., Cajaraville M.P., Angulo E., Giamberini L. (2002). Cellular and Subcellular Distribution of Metals in Molluscs. Microsc. Res. Tech..

